# Treatment of Dental Pulp Preparatory to Plugging

**Published:** 1849-10

**Authors:** J. D. White

**Affiliations:** Dentist.


### ARTICLE V.

*Treatment of Dental Pulp preparatory to Plugging.* By DR. J. D. WHITE, Dentist.

MR. EDITOR:—There is no subject connected with the duties of the dental practitioner so important as the above, and none which the writer would approach with more deference to the opinions of others. That the subject is intricate, all will agree; and that nothing has been settled upon, to direct the young practitioner in a way by which he may generally arrive at very satisfactory results, is also true. It is justly remarked by Mr. Tomes, of London, "that it is too much the practice, at the present day, to immediately remove an aching tooth. It would well repay any one who has time and opportunity, to devote their energies to the investigation of this subject," and that "there are many teeth extracted, which, with care, might be saved and rendered serviceable for years." The same remarks will apply, to a great extent, with reference to the subject in this country. Professor Harris, of Baltimore, remarks, in his work on Dental Surgery, published some time ago, that "Indeed, I regard the propriety of plugging a bicuspid or molar, after the nerve has been exposed, as so extremely doubtful, that I think I hazard nothing in asserting, that however correct the preparatory treatment may have been, it will not be successful in more than one case out of four." And more recently he remarks, in an article on the treatment of the pulp, in the American Journal and Library of Dental Science, July, 1849, that "even now, although he has performed the operation successfully in numerous instances, he feels considerable hesitancy with regard to the propriety of expressing his views upon the subject; nor would he at this time, had he not been frequently requested to do so." This eminent author further remarks, "although he is disposed to think favorab'y of it at present, its ultimate value, to some extent, remains to be determined;" but, "hereafter, he may furnish the readers of the Journal with the result of his observations and experience upon the subject." This is the right spirit. Combined observation is the only sure way by which we can hope to arrive at correct conclusions in any difficult subject. If such had been the practice of those illustrious names who have gone from among us, and a correct record handed down to the rising generation of the profession, incalculable good might have been done, for some of the ills that flesh is heir to, and an important work have been done, and fixed upon established principles, which, as yet, has scarcely been commenced. With regard to the propriety of attempting to treat the dental pulp, as a general rule, when exposed by decay, there can be no doubt. Subject, however, to many considerations, the age of the patient, as to whether the roots of the teeth are fully formed, as well as the general health and tone of the teeth, gums, and the system generally. But experience can only be rendered advantageous, in this respect, by close observation, founded upon an extensive knowledge of physiological and pathological science. However uncertain the treatment may be, it is better to make the trial, for even if the tooth is lost, it is no more than would happen at any rate, as the tooth is useless with an exposed pulp, and better learn by losing hundreds, than to abandon forever the attempt to preserve any. The writer has been making extensive experiments in the treatment of the exposed pulp, for twelve years in every conceivable way, and has finally settled upon a general and very successful plan of practice, and which plan he gave in full in a thesis paper on the treatment of the dental pulp preparatory to plugging, for the degree of Doctor of Medicine, in the Jefferson Medical College, in 1844, and which will form the basis of the present series of papers upon the above subject.

To better understand the subject, a few remarks, with reference to the division of tooth-ache into different stages, and the diagnosis only, will not be out of place, as it is presumed that students become, at the present day, acquainted with the minute anatomy, structure and physiology of the teeth, in the earliest part of their studies; those that have not, I would refer to *Tomes, Harris, Goodsir and others.* There is no case of toothache that cannot be cured, and the tooth saved, as a general rule, if there be enough of the dentine or body and root of the tooth remaining to receive a plug. Tooth-ache may be divided into, and treated under three heads, viz. *True, False* and *Sympathetic;* but may also be considered as only different stages of the same disease; because it is evident, that however remote or obscure the pain and pathological changes may be, if excited by a tooth, it is none the less tooth-ache in some of its forms or stages.

1st. *True tooth-ache* is acute inflammation of the dental pulp or nerve of the tooth only, and subject to the same changes as any other vascular tissues of the body, while running the different stages of inflammatory action, and the intensity and character of the pain depending somewhat upon, and marking the different pathological changes the pulp is undergoing at the time. *Its causes*—may be *constitutional, remote, approximate* or *local.* Constitutional, such as high sensibility and irritability of the nervous and vascular system. Remote, when other diseases are operating upon the system; such as tuberculous diseases of the nervous system, genital organs, attacks of cold, &c.; in short, any disease which operates to promote irritability and a morbid condition of the system, will favor an attack of the tooth-ache of any kind. Approximate and local; such as one diseased tooth operating upon another, by *metastasis,* sympathy or close proximity; decay of the dentine sufficiently to expose the pulp to air, and the irritating acids of the mouth, sudden and extreme changes of temperature, erosion, &c.; dead dentine without much softening, acting as a foreign substance, as in cases of blackness of the tooth-substance, commonly called black decay; on the contact of any foreign substance or plugging material, while introducing a plug; accumulation of serum, blood or pus beneath a metallic plug, or the decay of the tooth itself; when inflammation attacks the pulp before the decay is removed sufficiently to allow of the escape of the accumulating fluids.

2d. *False tooth-ache* is an inflammation of the alveolo-dental membranes and gums, and is commonly communicated from *within* the tooth to *without*, by continued inflammation and ulceration of the pulp, through the foramen at the end of the root; hence it almost invariably commences at the apex of the fang. This membrane never continues acutely inflamed for any length of time, without destroying the vitality of the pulp, because the swelling of the coats of the blood vessels around the foramen at the end of the root, cuts off a supply of blood to it, and the high grade of inflammation which exists in the pulp before it extends to any height externally, will cause it to slough. This is the point at which true alveolar abscess commences, and is never established without a loss of the dental pulp. Its causes, salivary calculi, (but, as observed above, generally diseases of the pulp,) which will often excite extensive inflammation of the gum and periosteal membranes, and sometimes to such an extent as to even inflame the pulp, and cause it to slough; a blow with any hard substance will often produce the same effect. Calomel is also a common cause of periosteal inflammation, especially when pushed to ptyalism, and acids of various kinds, administered during illness, and the mouth not washed carefully. But the most marked cases of the kind, and the most painful, but without the extreme sponginess which exists in severe ptyalism, that we have ever seen, has been during the development and eruption of the wisdom teeth, in patients of extreme irritability of the nervous and vascular system. And what is most curious, however loose, and however sensitive the teeth may become in ptyalism or teething, as soon as the irritating cause is removed, the teeth return again to their natural and healthy condition, as a general rule, without a loss of the pulp.

3d. *Sympathetic tooth-ache.*—This character of tooth-ache may be regarded as only existing in sound teeth, or in teeth in which pain is experienced, but are not themselves the exciting cause of the pain, but excited by some irritating cause along the course of the nerves of the same side of the face; not, as is supposed by some, caused by a diseased tooth of the same class on the opposite side. Opposite jaws may be painful from the same cause, but not opposite sides of the face, except it be from disease of the roots, or both of the nerves of the fifth pair—such as in rheumatism or irritability of the nerves of the head and face generally.

*Its causes.*—Diseased neighboring teeth; diseases of any character involving the fifth pair of nerves; general irritation of the gums from salivary calculi; partially necrosed roots; uterine pregnancy; development and eruption of the teeth; exostosis of the roots and alveolar processes; ossification of pulp, &c., &c.

*Diagnosis of true tooth-ache.*—Actual contact with your instrument, after removing the decay of the tooth, and ocular demonstration, are almost the only positive signs of tooth-ache; still the following symptoms may sometimes lead to correct conclusions, viz. pain upon taking substances into the mouth above or below the common temperature of the blood. Yet high sensibility of the tooth, when only slightly decayed, or where they are wholly sound, may give rise to great pain upon taking cold or sweet substances into the mouth; and sometimes cold is the only temporary remedy for inflamed pulp; therefore, a tooth-ache which is relieved by cold water, may be relied upon as arising from inflammation of an exposed pulp; on the contrary, warm, when it produces any impression at all, it is to increase the pain, and that is frequently the first sign we have of the inflamed pulp, after a tooth has been plugged with slight exposure of the nerve. Tenderness of the tooth *inside* of the *cavity of decay*, and more or less prolonged pain after the instrument is removed; while pain excited by sensibility of the bone, only lasts while the instrument is in actual contact with it. Again, a little experience will render the operator capable of judging whether the pain, excited by the contact of his instrument, is really from an exposed pulp or sensitive bone, by the peculiar thrill which it gives the patient.

These symptoms all become much exalted when acute inflammation attacks the pulp, together with intense pain accompanying. Intermitting pain is also a marked sign of true tooth-ache, especially in the after part of the day, and fore part of the night—the febrile exacerbation—the determination of blood to the head and face, which gives the flushed cheek more or less to all in the evening, accounts for more pain being experienced at this time than any other in the twenty-four hours. Few have tooth-ache in the morning; hence, the promises which are made in the night, that the tooth shall be extracted in the morning, are, on account of the absence of pain at that time, so frequently broken by the sufferer. When these symptoms are present, and there is no seeming elongation of the tooth from the socket, and no undue sensation by sharply striking against the cutting edge or grinding surface of the tooth, with a hard instrument, it may be generally relied on as diagnostic of true tooth-ache.

[*Dental News Letter.*

### Miscellaneous Notices.

